# Lack of Adjustment Latitude at Work as a Trigger of Taking Sick Leave—A Swedish Case-Crossover Study

**DOI:** 10.1371/journal.pone.0061830

**Published:** 2013-04-19

**Authors:** Hanna Hultin, Johan Hallqvist, Kristina Alexanderson, Gun Johansson, Christina Lindholm, Ingvar Lundberg, Jette Möller

**Affiliations:** 1 Department of Public Health Sciences, Karolinska Institutet, Stockholm, Sweden; 2 Department of Public Health and Caring Sciences, Uppsala University, Uppsala, Sweden; 3 Department of Clinical Neuroscience, Division of Insurance Medicine, Karolinska Institutet, Stockholm, Sweden; 4 National Centre for Work and Rehabilitation, Department of Medicine and Health Sciences, Linköping University, Linköping, Sweden; 5 Department of Medical Sciences, Division of Occupational and Environmental Medicine, Uppsala University, Uppsala, Sweden; Catholic University of Sacred Heart of Rome, Italy

## Abstract

**Objectives:**

Research has shown that individuals reporting a low level of adjustment latitude, defined as having few possibilities to temporarily adjust work demands to illness, have a higher risk of sick leave. To what extent lack of adjustment latitude influences the individual when making the decision to take sick leave is unknown. We hypothesize that ill individuals are more likely to take sick leave on days when they experience a lack of adjustment latitude at work than on days with access to adjustment latitude.

**Methods:**

A case-crossover design was applied to 546 sick-leave spells, extracted from a cohort of 1 430 employees at six Swedish workplaces, with a 3–12 month follow-up of all new sick-leave spells. Exposure to lack of adjustment latitude on the first sick-leave day was compared with exposure during several types of control periods sampled from the previous two months for the same individual.

**Results:**

Only 35% of the respondents reported variations in access to adjustment latitude, and 19% reported a constant lack of adjustment latitude during the two weeks prior to the sick-leave spell. Among those that did report variation, the risk of sick leave was lower on days with lack of adjustment latitude, than on days with access (Odds Ratio 0.36, 95% Confidence Interval 0.25–0.52).

**Conclusions:**

This is the first study to show the influence of adjustment latitude on the decision to take sick leave. Among those with variations in exposure, lack of adjustment latitude was a deterrent of sick leave, which is contrary to the à priori hypothesis. These results indicate that adjustment latitude may not only capture long-lasting effects of a flexible working environment, but also temporary possibilities to adjust work to being absent. Further studies are needed to disentangle the causal mechanisms of adjustment latitude on sick-leave.

## Introduction

Adjustment latitude is defined as the possibility to temporarily adjust one's work demands to the loss of function due to illness or disease [1], [2]. In their Illness Flexibility Model (IFM), Johansson and Lundberg state that adjustment latitude affects the extent to which a loss of function affects an individual's work ability [2]. Access to adjustment latitude differs between socioeconomic groups, occupations and workplaces due to the nature of the work tasks performed, but also likely due to the organizational culture [1]. A low general level of adjustment latitude, measured once and considered stable over time, has been associated with sick leave in a cohort study based on the same research project as the present study and in two other studies [1], [2], [3]. When an individual is ill, the use of adjustment latitude can constitute an alternative coping strategy to sick leave [1], [2], [4], [5].

Adjustment latitude is expected to affect sick leave by letting individuals adjust work when they are ill, thereby decreasing their need to take sick leave. Lack of adjustment latitude then becomes a risk factor that can trigger the decision to take sick leave when ill [1], [2], [3]. Previous studies suggest that many individuals experience variations in access to adjustment latitude, [2], [3], [6] but the time interval between exposure and outcome has not been considered when analyzing the association between adjustment latitude and sick leave [1], [2], [3]. The case-crossover design is a study design aiming to identify triggers and quantifying their effect [7], [8]. We have used this design to study potential triggers of taking sick leave, and have in previous studies identified problems in relationships with colleagues and superiors and both high and low workload as triggers of sick leave [9], [10]. The hypothesis in this study is that ill individuals are more likely to take sick leave on days when they experience a lack of adjustment latitude at work, than on days with access to adjustment latitude.

## Materials and Methods

The TUFS project (an acronym for “triggers of sickness absence” in Swedish) was carried out at six workplaces, located at geographically disparate areas in Sweden, between April 2005 and February 2007. The project was designed as a case-crossover study nested within a cohort. The project has been reviewed by the Regional Ethic Review Board in Stockholm and conforms to the principles of the Declaration of Helsinki. The project was approved by Stockholm's Regional Ethic Review Board. The cohort included the 1 430 employees (47% of the study population) who agreed to participate by returning a baseline postal questionnaire and a consent form.

### Study design

In a case-crossover study, the exposure frequency during a time period close to the outcome, the case period, is compared to the exposure frequency during control time periods within the same individual [7], [8]. In this study, exposure to lack of adjustment latitude during periods of sick leave was compared to exposure during periods when the individual was not on sick leave.

**Figure 1:**
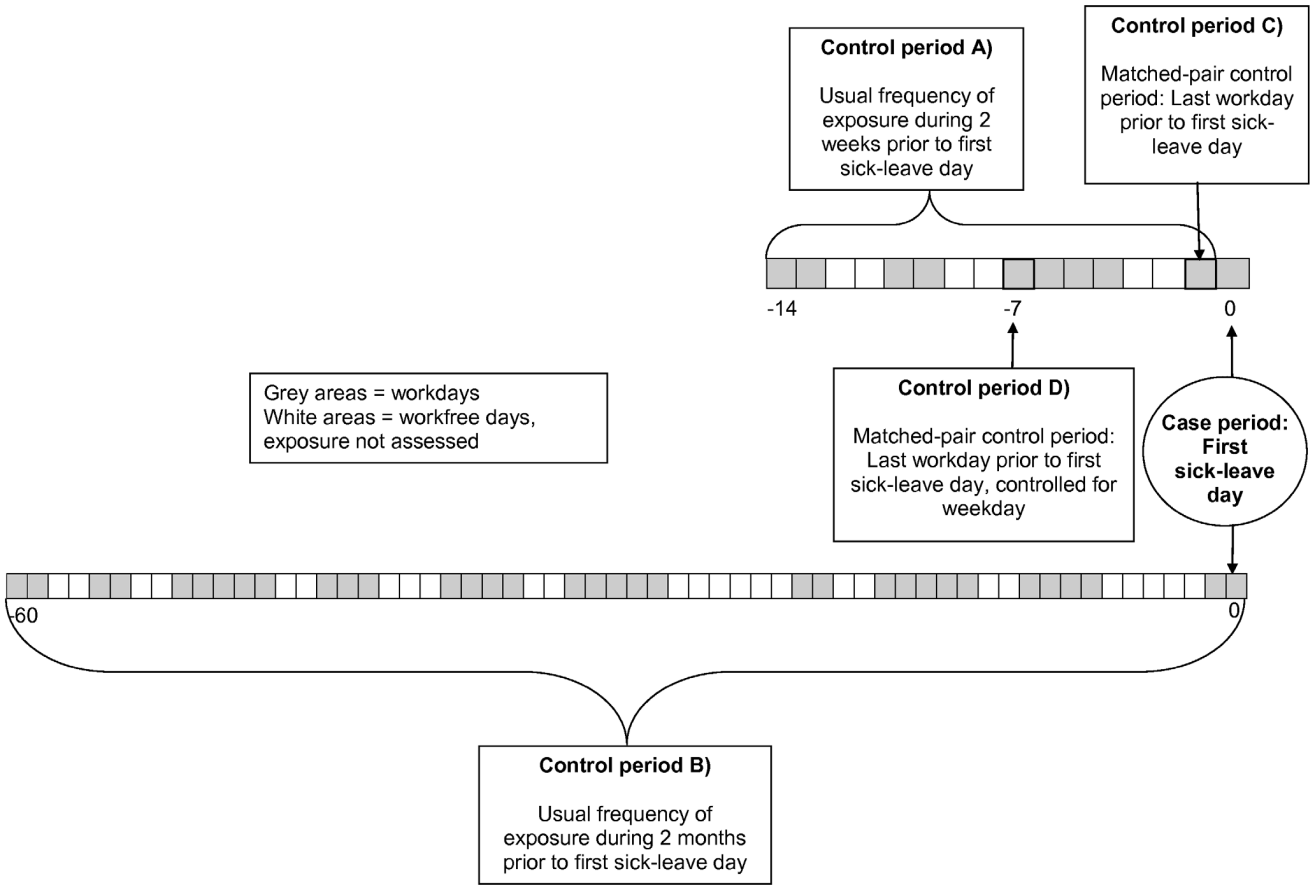
Description of the case and control time periods used in the analyses. The case-crossover design of the study implies analyzing exposure in different case and control time periods for the same individual. The figure describes the case period and the four alternative control periods used in the analyses.

The case period was defined as the first sick-leave day. If a respondent worked part of a day and then took sick leave, that day was defined as the first sick-leave day. Four different control time periods were defined (see Figure 1): (a) A usual frequency of exposed workdays, based on a two-week period prior to the first sick-leave day, (b) a usual frequency of exposed workdays during the two months prior to sick leave, (c) a matched-pair control period corresponding to the last workday before the first sick-leave day, and (d) a matched-pair control period corresponding to the last workday before the first sick-leave day, controlled for weekday.

### Source population

We contacted workplaces strategically chosen to cover different occupational sectors. Six Swedish workplaces with 30–1 200 employees participated, four public or municipal health-care facilities, one private manufacturing plant, and one private insurance company. The major occupational groups at the workplaces were nurses and assistant nurses at the health-care facilities, process operators and machine operators at the factory, and insurance specialists and insurances sales persons at the insurance company.

Human resource staff at the respective workplaces identified 3 020 employees with a contract for more than three months future employment, who were neither on parental leave, sick leave for more than 30 days, nor other leave of absence, and these were invited to participate. The cohort included 1 430 employees (47%).

Data from three sources were obtained; 1) a baseline questionnaire, including questions on health, private life and work environment, 2) daily information on the start and end dates of all new sick-leave spells and 3) telephone interviews containing trigger exposure information, conducted as soon as possible after the start of the sick-leave spell.

### Sick-leave spells

**Figure 2:**
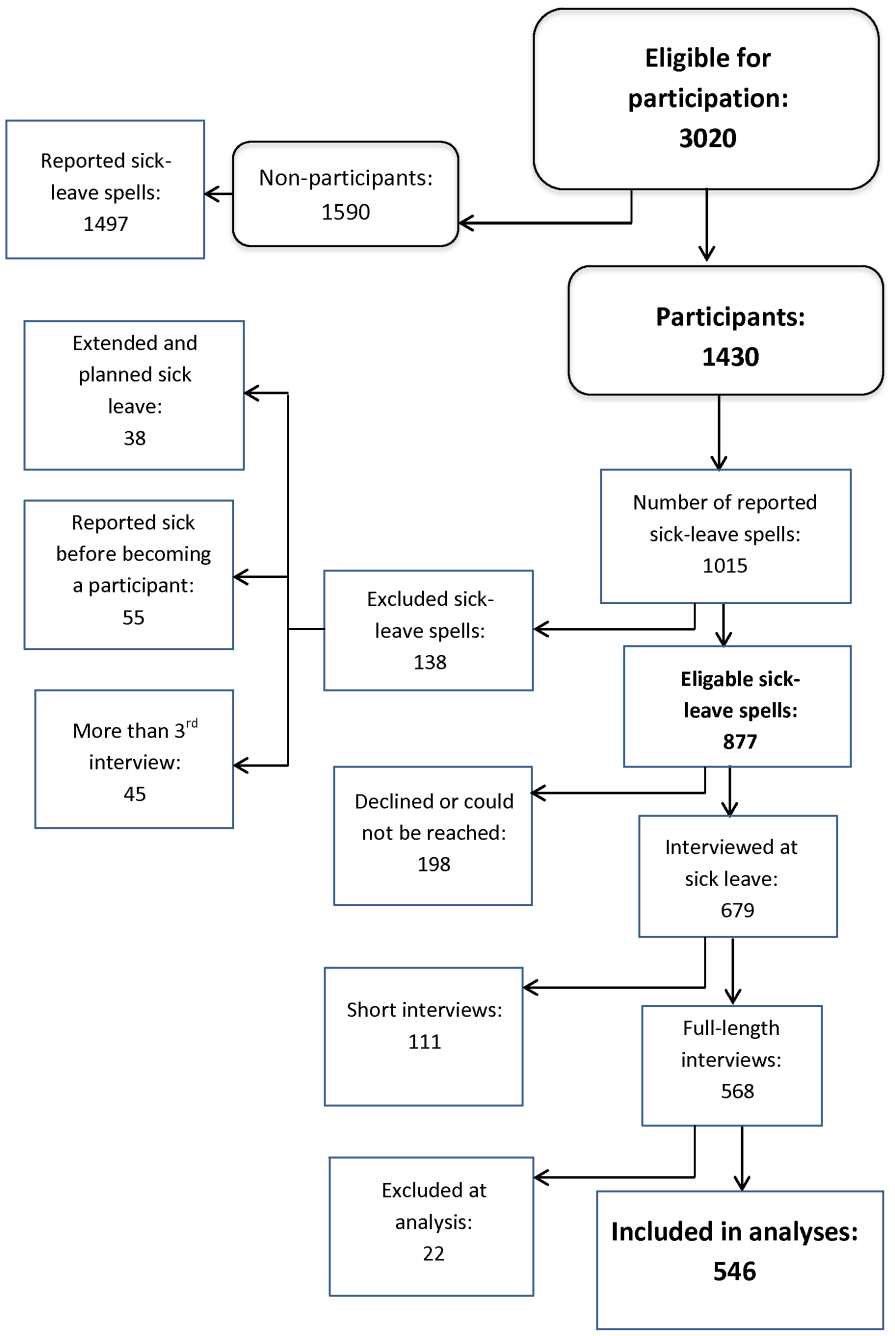
Flow chart of the data collection of sick-leave spells. Description of inclusion and exclusion of sick-leave spells in the present study, derived from the 1, 430 participants of a cohort study carried out at six Swedish workplaces between 2005 and 2007.

A sick-leave spell was defined as each time a participant reported sick to the workplace. These were identified through daily reports by email or fax from the workplaces. All sick-leave spells for a 3–12 month follow-up were considered eligible, except planned sick leave (i.e. for planned surgery). Overlapping or extended spells were considered as one spell. The unit of analysis is sick-leave spells, and some individuals contribute with more than one spell; however with different case and control periods. Except for an initial pilot period, individuals who had participated in three interviews were not contacted again (45 spells were excluded for this reason), leaving a total of 877 eligible sick-leave spells (see Figure 2).

Out of the total 877 eligible sick-leave spells, an interview was conducted in 679 spells (mean time from first sick-leave day to interview = 2 days). In 198 (23%) of the spells the absentee declined interview or could not be reached. In 111 (16%) of the interviewed spells, a shortened version of the interview, with no exposure information was conducted. This was done mainly when the absentee did not have time or strength to complete a full interview and where the prospect of such was judged small. Furthermore, 22 interviews, in which more than 14 days had passed between the first sick-leave day and the interview, were excluded to enhance data quality. Hence, the total number of analyzed sick-leave spells was 546 (62% of eligible sick-leave spells) originating from 432 individuals. Characteristics of the participants are presented in Table 1. More details on the data collection have been published previously [3], [9].

**Table 1:** Background characteristics of interviewed sickness absentees.

**Background characteristics**		n = 432
**Age**	Mean	43,06
	Standard deviation	11.34
	Maximum	66
	Minimum	20
**Sex**	Women	60.70% (261)
**Occupational sector**	Health care	22.69% (98)
	Factory	53.70% (232)
	White collar workplace	23.61% (102)
**Socioeconomic status**	Unskilled manuals	2.35% (103)
	Skilled manuals	19.86% (84)
	Lower non-manuals	20.57% (87)
	Middle non-manuals	30.50% (129)
	Higher non-manuals	4.73% (20)
**Self-rated health**	Very good or good	77.96% (336)
	Fair, poor or very poor	22.04% (95)
**Housework**	All (100%)	23.80% (99)
	Half or more	59.38% (247)
	Less than half	16.83% (70)
**Partner relationship**	Yes	77.46% (330)
**Have children (age 0–18 yrs.)**	Yes	44.37% (189)
**Attendance requirements**	Always or often	9.52% (40)
	Sometimes	40.24% (169)
	Seldom or never	50.24% (211)
**Adjustment latitude, general (at baseline)**	Never	27.87% (119)
	Seldom	25.76% (110)
	Sometimes	36.77% (157)
	Often	9.60% (41)
**Number of sick-leave days during the 12 months prior to inclusion**	None	16.98% (91)
	1–7 days	52.80% (283)
	8–30 days	24.25% (130)
	31–90 days	2.05% (11)
	More than 90 days	3.92% (21)

### Exposure

All exposure information was collected through the telephone interviews at sick leave. Two types of adjustment latitude were assessed, “general adjustment latitude” and “health-problem dependent adjustment latitude”. Both types of adjustment latitude were measured through a set of questions lead by a gate question. For general adjustment latitude the question read: “During the last year, have you had possibilities to change your work tasks or your workday in any of the following ways, if you would have needed to for some reason?”. Following this question, six different types of adjustment latitude were enumerated by the interviewer (postpone work, change work tasks, work slower, take longer breaks, shorten the workday or work from home). This definition was based on previous research [1], [2]. For health-problem dependent adjustment latitude the question read: “Was there any day during the last year during which you could have changed your work tasks or your workday to such an extent that you would have been able to work despite having [the reported health problem] such as the one you had on [the first sick-leave day]?”.

Respondents reporting no access to adjustment latitude during the last year were considered exposed to lack of adjustment latitude all the time. If the respondents answered “yes” to the gate question, they were further asked to estimate the number of workdays with access to any of the respective types of adjustment latitude during the two months prior to sick leave, and for each specific workday of the two weeks prior to sick leave. Finally the respondents were asked whether they would have had access to adjustment latitude on the first sick-leave day if they would not have been on sick leave.

The exposure status in the case period was based on information concerning the first sick-leave day. All workdays without adjustment latitude in the two-week period before sick leave were summed into a two-week usual frequency (Figure 1: control period a). The two-month usual frequency (Figure 1: control period b) was calculated by subtracting the number of workdays with access to adjustment latitude from baseline information on the number of normal monthly workdays. The matched-pair control periods (Figure 1: control periods c and d) were also extracted from the two-week period.

Respondents who were unable to answer the gate question, or to estimate their adjustment latitude in the case or control periods were excluded from those respective analyses. In total, 452 spells (83% of all spells in the study) contained information on general adjustment latitude in the case period and at least one control period. The equivalent number for health-problem conditional adjustment latitude was 467 (86%). If the respondent was unable to pinpoint the specific day during the two-week period which was exposed this was considered as “uncertain exposure” and given a special code.

### Effect modifiers

Data on age, sex, self-rated health, having a partner, having children, share of housework, baseline adjustment latitude, attendance requirements, and sick-leave history was extracted from the baseline questionnaire for descriptive and effect modification analyses. Self-rated health was measured by the question: “What do you consider your health status as in general?” with five answer alternatives (Very good, Good, Fair, Poor and Very poor). Share of housework was measured through the question: “How large a share of the total amount of house work do you perform?”, and the reported percentages were divided into “All”, “Half or more” and “Less than half”. Attendance requirements was assessed through the question: “Can staying home for one or two days because of illness, be hard for you because of work?”, with three answer alternatives (“Always or often”, “Sometimes”, and “Seldom or never”). Baseline adjustment latitude was measured through the question: “If you are tired, out of sorts, or have a headache, are you able to adjust work to how you are feeling?” with the answer alternatives “Never”, “Seldom”, “Sometimes” and “Often”.

Occupational titles from the questionnaire were coded into 2-digit SEI codes (Socioeconomic Classification) [11]. Sick-leave duration was calculated from the start and end dates of the reported sick-leave spells, however without excluding work-free days within a spell, and categorized into ≤7 days and ≥8 days.

When interviewed, the respondents were asked about their own opinion about the reason for taking sick-leave. These self-reported health problems were coded into eight broad categories. The respondents were also asked whether there were any circumstances besides illness which influenced their decision take sick leave. These answers were coded into “work related” and/or “private related”.

### Statistical analysis

Odds ratios (OR) were calculated using Mantel-Haenszel estimators with confidence intervals (CI) for sparse data, and by conditional logistic regression [7], [8], [12], [13]. Each sick-leave spell, with case and control periods, was considered as one stratum in the analyses. The odds ratios are considered as estimates of the incidence rate ratio comparing exposed to unexposed conditions [8], [14], [15].

Effect modification by stable factors was investigated by stratifying the analyses employing the two-week usual frequency control period by sex, occupational sector, socioeconomic position, length of sick-leave spell, baseline adjustment latitude, and attendance requirements.

Several different sub-analyses were conducted for the purpose of data quality control. These included coding uncertain exposure events as missing, using a more strict restriction criteria regarding time between reported sick leave and interview, analyses restricted to first-time interviews, analyses excluding sick-leave spells where the respondent had worked part of the first sick-leave day (12%), and analyses stratified by interviewer.

## Results

Thirty-nine percent of the participants in the cohort had recorded sick-leave spells during the follow-up. The duration of the sick-leave spells were generally short, 86% of the spells included in the study lasted less than 8 days. The self-reported health problems were in a majority of the spells related to minor infections, colds and influenzas (data not shown).

The most common type of adjustment latitude reported in the interview was to shorten the workday, which 75% of the employees reported having during the last year. Only 11% reported being able to work from home during the previous year. In 8% of the spells, the employees reported not having had access to any type of adjustment latitude during the last year. Twenty-nine percent reported lack of general adjustment latitude on the first sick-leave day and 92% reported lacking adjustment latitude enough to adjust work to their health problem that day. A large proportion of the respondents reported a stable pattern of exposure, i.e. either being constantly or never exposed, 46% (209) reported never being exposed to lack of general adjustment latitude in the previous two weeks, 19% (84) reported being constantly exposed during the two weeks, and 35% (159) reported a varying frequency of exposure over the two-week period.

Table 2 shows that the risk of sick leave on a day with lack of general adjustment latitude at work is lower than on a day with access to adjustment latitude, with an OR of 0.36 (95% CI 0.25–0.52) when using a two-week usual frequency control period. For all four control periods, the results are similar. Lack of health-problem dependent adjustment latitude also resulted in effect estimates below 1 (OR = 0.04 95% (CI 0.01–0.16) for two week usual frequency, and OR = 0.13 95% (CI 0.05–0.33) for two month usual frequency.

**Table 2:** Odds ratios of sick leave on a day exposed to lack of adjustment latitude, relative to an unexposed day, with surrounding 95% confidence intervals.

Analytic approach	Type of control information	Odds ratio	95% confidence interval
Usual frequency	The two week period prior to sick leave	0.36	0.25–0.52
Usual frequency	The two month period prior to sick leave	0.34	0.23–0.48
Matched pair^1^	The last workday before sick leave	0.42	0.26–0.68
Matched pair^2^	The last workday before sick leave controlled for weekday	0.41	0.25–0.65

1 There were 97 cases who reported lack of adjustment latitude in both the case and the control period. Fifty-seven cases were exposed only during the last workday before sick leave, as compared to 24 cases who reported exposure only during the first sick-leave day.

2 There were 77 cases who reported lack of adjustment latitude during both the case and control period. Fifty-nine cases reported lack of adjustment latitude only during the last workday before sick leave that was the same weekday as the first sick-leave day, as compared to 24 cases who reported only being exposed at the first sick-leave day.

Stratified analyses indicated that the deterring effect of lack of adjustment latitude was similar for men and women, all occupational sectors, all sick-leave spell lengths, and all baseline levels of attendance requirements and adjustment latitude (data not shown). Similar effects were present in all socioeconomic groups, except among “higher non-manuals”, where no deterring effect could be seen (data not shown). We also performed analyses stratified by the self-reported reduction of work ability at sick leave. In the group who reported the smallest reduction in work ability at sick leave (50–99% of normal work ability) a slightly stronger deterrent effect of exposure was suggested (OR 0.09 CI 0.02–0.36).

The different analyses made for the purpose of quality control showed no marked changes in estimated effects.

## Discussion

The hypothesis was that the effect of low level of adjustment latitude on sick leave operates by implying a lack of adjustment latitude when needed, i.e. when being ill. Our findings indicate the opposite; lack of adjustment latitude appears to be a deterrent of sick leave rather than a trigger. This implies either serious bias, or that the assumed mechanism of adjustment latitude is more complex than assumed.

However, 65% of the cases reported no variation in exposure, thereby not contributing information to the analysis. The most striking feature of lack of adjustment latitude is the high proportion of constantly exposed respondents (19% during the two weeks prior to sick leave) and it seems very reasonable to assume that among this group, the exposure to lack of adjustment latitude at the time of taking sick leave contributed to their sick leave, as suggested in previous cohort studies [1], [2], [3]. However, this cannot be revealed in a case-crossover analysis.

Descriptive analyses did not reveal any apparent differences between the groups with constant and time-varying exposure. Among those with variations in exposure the deterrent effects were also found among both sexes, in all occupational sectors, and among all socioeconomic groups but one.

The sick-leave spells in our data mainly consist of short-term absences for different types of acute infections, and it is quite probable that rather extensive adjustment possibilities are needed to adjust work to such health disorders. For a large group such possibilities may never exist. In light of that, one possible interpretation is that although many respondents reported access to some adjustment latitude on the first sick-leave day (71%), very few respondents had enough adjustment latitude to adjust work to their health disorder (8%). Still, although many individuals were exposed in the case period, exposure had a deterrent effect.

### Confounding

It is possible that certain adjustment possibilities are presented to an individual only when ill. This implies an apparent risk of confounding from illness, causing an increased frequency of adjustment latitude (i.e. non-exposure to lack of adjustment latitude) in the case period only, leading to decreased risk estimates. The two different definitions of adjustment latitude were used to handle this potential problem. The health-problem conditional definition of adjustment latitude resulted in fewer individuals being exposed in the case period as well as in the control periods, which is expected, but the effect estimates were still below one. This indicates that even when trying to keep the severity of illness constant in the case and control periods, cases were more likely to have taken sick leave on a day with access to adjustment latitude.

Another possible explanation may be co-varying patterns of other triggers or deterrents of sick leave. One such possible confounder is the workload, since a low level of adjustment latitude also may indicate a high workload, which may act as an incentive to attend work. In the interview, respondents also reported their day-to-day exposure to “a very stressful work situation, indicated by more work tasks, less personnel or larger area of responsibility than usual”. We performed an analysis, using control period c (Figure 1), of “lack of adjustment latitude” adjusting for simultaneous exposure to “a very stressful work situation”. This resulted in very small changes in the effect estimates for lack of adjustment latitude (OR 0.45 CI 0.30–0.68). In fact, we have shown that exposure to “a very stressful work situation” has a triggering rather than a deterring effect on sick leave [9].

The mechanism of adjustment latitude may differ between different work contexts with different absence cultures (which may mandate the agreed upon level of sick leave and reasons for sick leave). This is included in the concepts “incentives” and “requirements” in IFM, which according to the model may mediate the effect of adjustment latitude on sick leave. One such concept, which is closely related to workload and to the decision to take sick leave when ill, is attendance requirements, which includes the negative consequences of absence for the individual, colleagues or a third party [2]. When one is absent, work tasks may pile up, colleagues may get burdened, and activities may get cancelled. Attendance requirements have been shown to be associated with both sick leave and adjustment latitude [2], [3]. Moreover, attendance requirements, like adjustment latitude, are likely to vary over time. Unfortunately, we do not have information on the day-to-day variation in exposure to attendance requirements. The stratified analyses of the baseline measure of attendance requirements showed no marked effect modification. However, the explanation to why our results differ from that of previous longitudinal studies [1], [3] could be that adjustment latitude captures more than one aspect of the work environment: it may capture long-lasting health effects of a flexible work environment, and among individuals with day-to-day variations in adjustment latitude, access to adjustment latitude on the first sick-leave day may also capture possibilities to adjust work to being absent when ill, i.e. a lack of attendance requirements. This last interpretation is further strengthened by our previous study which indicated that an increased risk of sick leave was found on days with a low workload [10].

### Information bias

A common approach to address information bias in case-crossover studies is to employ different types of control information and compare the results [16]. We used four different control periods from two types of control information, all resulting in similar effect estimates. To avoid attribution of exposure to certain time periods, neither the interview subjects nor the interviewers were informed about the hypothesized hazard period.

The fact that respondents were on sick leave when interviewed may have affected their estimation of exposure in the case periods. If the respondents over-reported lack of adjustment latitude in the case period in order to justify their absence, this would cause an overestimation of the OR. To explain our results, respondents would instead have to under-report lack of adjustment latitude in the case period. We find this to be unlikely.

Misclassification of exposure in the control periods may also be a possible explanation of the results. To explain the deterrent effect, the respondents would have to systematically under-report access to adjustment latitude in the control periods. This could be due to general recall bias, since the control periods are further away in time than the case period. This problem should increase the longer the control period and the further away from the case period the control period is situated. The similar effect estimates from all analyses, whether based on the workday immediately before the case period or on two months before the case period, implies that such bias are limited. Furthermore, when sub-analyses were made with different restrictions on the allowed time interval between reporting sick and completion of the interview, this only resulted in minimal changes of the effect estimates.

### Generalization

The strategic sampling process, together with the non-participation on cohort and interview level, has to be considered when generalizing the results. It is also important to note that a rather large proportion of our sample was constantly exposed to lack of adjustment latitude, and we cannot assume that our results can be generalized to that group.

Of the 3 020 individuals eligible for participation, 53% declined to participate. The estimated sick-leave incidence was 4.30/1 000 person-days among those declining participation, compared to 2.85/1 000 person-days among the participants. However, to imply selection bias, case selection (i.e. participation) would need to be related exposure in the case period, and we have no reason to believe that non-participants who reported sick during the follow-up are more or less likely to have been exposed to lack of adjustment latitude when reporting sick.

The case-crossover design implies that employees who did not have any sick-leave spells during follow-up (61% of the cohort participants) are not included analyses. Baseline information shows that this group includes more men, more individuals with non-manual occupations, with better self-rated health and more general access to adjustment latitude than those included in the analyses (data not shown). The exclusion of non-cases is not a methodological limitation, but rather and effect of the differing operational hypotheses of case-crossover studies and traditional cohort and case-control studies [17]. Whereas a cohort or case-control study may aim to answer the question “Why did these people take sick leave?” our study aims to answer the question “Why did these people take sick leave now? What is different about this day?” It is not part of our research question to explain why certain individuals did not report sick during the follow up.

Of the 877 eligible reported sick-leave spells, 40% were for different reasons not included in the analyses. This might imply a selection of sick-leave spells, or absentees, with certain characteristics. Comparisons between the sick-leave spells which were included and those where the absentee declined to be interviewed, revealed no differences with regard to age, sex of the absentee, nor with regard to their occupational sector. Furthermore, no statistically significant differences in baseline levels of adjustment latitude between the included sick-leave spells and the excluded spells were found. Comparisons between included sick-leave spells and spells in which only a short interview was conducted did not reveal any differences in self-rated work ability at sick leave or self-reported reasons for sick leave. In total, 52% the individuals who declined an interview or could not be reached were included in the study with another sick-leave spell.

Since some individuals were interviewed more than once, as 432 individuals contributed to the 546 sick-leave spells. This dependency may slightly underestimate the variance and may possibly affect the risk estimates [18]. However, the exposed sick-leave spells are not confined to spells from a small selected group of individuals, and when restricting the analyses to first-time interviews, the results only changed minimally.

## Conclusions

Our study showed that only 35% of the participants reported day-to-day variation in exposure to lack of adjustment latitude. For those that did report variation, lack of adjustment latitude at work appeared to have a deterring effect on sick leave. We have not found any obvious methodological deficiency that would explain this finding, which is contrary to our hypothesis. Our findings regarding the effects of short-term variations in adjustment latitude adds complexity to the mechanisms through which adjustment latitude affects the levels of sick leave. In light of previous research, the current results indicate that adjustment latitude may not only capture long-lasting effects of a flexible working environment, but also temporary possibilities to adjust work to being absent. Further research needs to investigate the associations between the day-to-day variations in adjustment latitude and attendance requirements.
